# Hourly Relationship between Reference Evapotranspiration and Shoot Shrinkage in Walnut Trees and Pomegranate under Deficit Irrigation

**DOI:** 10.3390/plants11010031

**Published:** 2021-12-23

**Authors:** Eduardo Salgado, Nieggiorba Livellara, Esteban Chaigneau, Fernando Varas, Italo F. Cuneo

**Affiliations:** Escuela de Agronomía, Pontificia Universidad Católica de Valparaíso, Quillota 2340025, Chile; nieggio@gmail.com (N.L.); esteban.chaigneau@gmail.com (E.C.); grifo_1@msn.com (F.V.)

**Keywords:** hourly shrinkage, hourly reference evapotranspiration, dendrometry, linear regression analysis, tree water relations, irrigation scheduling

## Abstract

Diameter fluctuations of branches, shoots, or fruits are related to plant transpiration and water potential. In the past, several models have related dendrometric variables and evapotranspiration on a daily scale. However, trunk–branch shrinkage occurs only between dawn and midday, while evapotranspiration occurs most of the day from sunrise to sunset. Previous models have failed to incorporate this key fact. The objective of the present study was to assess the relationship of hourly daily shrinkage (HDS) between dawn and the next 4 h to the hourly reference evapotranspiration (EToh) of the same period in walnut trees and pomegranate plants under different irrigation regimes. Our data show that the relationship between EToh and HDS is much better than several previous models that included maximum daily shrinkage (MDS) and reference evapotranspiration (ETo). The novel slope analysis of the relationship between HDS versus time used here corresponds to the velocity at which the HDS occurs, which depends on the ETo intensity at that moment. This new method of analyzing this type of data calls for a revision of these models and sets a new baseline for future analysis.

## 1. Introduction

When plant transpiration begins at dawn, the water required to sustain the transpiration stream comes from the internal organs and tissue of the plant itself [1]. Different storage compartments, such as dead fibers, nonfunctional vessels, and apoplastic pores, provide the largest fraction of stored water in most trees [2,3], but independently of specific anatomies of different species, the water held in these compartments can represent up to 50% of total water transpired in a single day [4]. When xylem compartments give up water to the transpiration stream, branches and shoots contract their diameter on a micrometric scale. The diameter recovering process begins when evapotranspiration intensity decreases and, therefore, roots can supply water to rehydrate xylem compartments [5]. Dendrometers can record these diameter fluctuations [6,7,8] and use these data for plant-based irrigation programming. Yet, the interpretation of radial stem variations in terms of growth and tree water relations in time series has been challenging due to the co-occurring processes: the irreversible stem expansion of growing cells and the reversible tree water deficit-induced shrinking and swelling of the stem [9,10]. Nevertheless, when data are analyzed in a daily time frame, the resulting information is valuable in assisting growers with irrigation scheduling decisions. 

**Figure 1:**
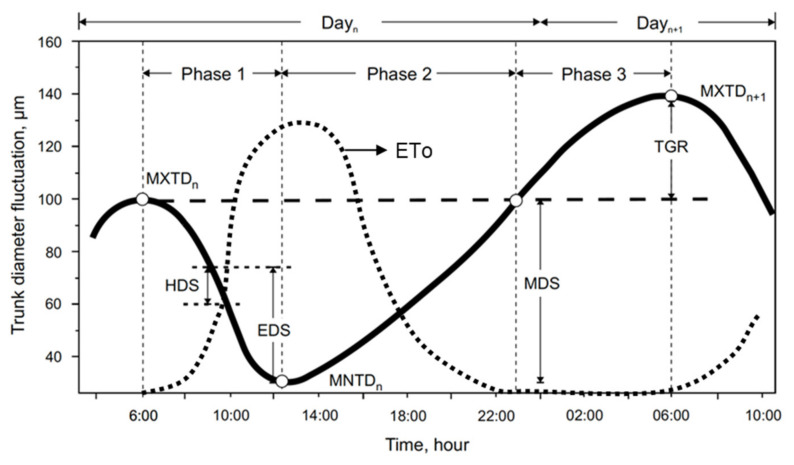
Schematic illustration of the typical daily shrinkage–recovering fluctuation process and its relationship with ETo. The figure displays different dendrometric variable definitions: MXTD, maximum daily diameter; MNTD, minimum daily diameter; MDS, maximum daily shrinkage; EDS, early daily shrinkage; HDS, hourly daily shrinkage; TGR, trunk growing rate. Phase 1 refers to the period between MXTD_n_ (day n) and MNTD_n_; Phase 2 refers to the period between MNTD_n_ and back to MXTD_n_; Phase 3 refers to the period between MXTD_n_ and MXTD_n+1_ (day n + 1).

Dendrometry data (i.e., daily branch diameter fluctuations) were divided into several variables in an effort to develop an indicator of plant water status. From maximum daily diameter (MXTD) and minimum daily diameter (MNTD) intervals, several variables were derived such as maximum daily shrinkage (MDS), early daily shrinkage (EDS), and trunk growing rate (TGR) (Figure 1). The variables found in the past that best represented plant water status were MDS [11,12,13] and TGR [6,14,15]. Some authors related EDS to reference evapotranspiration (ETo) [11,13,16] and to stem water potential (*Ψ*_stem_) [17,18]. Thus, reports show this variable as a good indicator of atmospheric demand and water stress [19]. However, not a single universal model exists relating ETo with dendrometry variables for all plant species and cropping conditions. The reason for this is that this relationship depends on the phenological phase of the crop [13,20], pathogen incidence [21], water stress [15], or meteorological conditions [22]. Several studies have reported on intra- and interspecies lack of consistency of dendrometry data for pomegranate [16,23], table grapes [24], cherries [25], kaki [26], tomatoes [27], apples [17], lemon [28], olives [29], plums [16], and almonds [30], thus producing a wide diversity of models for each species. In addition, these previous studies showed that ~40% of stem diameter fluctuations were not related to the corresponding evapotranspiration, probably because the relationship might require nonlinear models or because these fluctuations are highly dependent on ETo intensity [25]. Searching for a more precise model, De la Rosa et al. [31] defined a new dendrometric variable, EDS (Figure 1), which considers data from 9:00 AM to 12:00 PM. They suggested this variable due to the substantial variability among plants observed when using MDS under particular conditions [31,32]. They concluded that EDS relates to *Ψ*_stem_ more tightly than MDS in a study conducted with peach trees. However, the method applied by these authors establishes a fixed period from 9:00 AM to 12:00 PM, without considering that the branch shrinkage depends on meteorological conditions. 

In the present study, we hypothesized that if the EDS data for just four hours (9:00 AM to 12:00 PM) relates well with *Ψ*_stem_, it is possible that EDS also relates well with ETo, obtaining a more precise model. Combining the model proposed by De la Rosa et al. [31] with a model to estimate daily ETo based on hourly ETo data [33], we aimed to study the relationship of the hourly branch shrinkage from dawn to the next four hours in walnut and pomegranate under different irrigation treatments with the hourly ETo in the same period.

## 2. Materials and Methods

### 2.1. Characterization of the Study Area

We conducted the study within Petorca, Chile, in walnut and pomegranate orchards (coordinates 6,408,823 S, 310,783 E for Walnut and 6,409,151 S, 31,1667 E for Pomegranates). Both orchards are in the “Valle Río Petorca-Cabildo” agroclimatic district [34] with an inner steppe arid climate, 274 mm of annual rainfall, and nine dry months from September to May. This agroclimatic district presents an average monthly temperature between 8 °C for the coldest month (July) and 21 °C for the warmest month (February). The average minimum temperature varies between 2 (July) and 14 °C (February). The average maximum temperature varies between 16 (July) and 30 °C (March). The annual accumulation of chilling hours is 550–800 between the months of May and July. The presence of frost events (temperatures below 0 °C) occurs between the months of May and August; however, 68% of them are concentrated in July. The annual accumulated average evapotranspiration (ETo) is approximately 1200 mm. The soil of the walnut orchard presented a clay loam texture, pH of 7.4, an electrical conductivity of 1.1 dS m^−1^, and 1.5% of organic matter. A concentration of 16 mg kg^−1^ of available nitrogen, 60 mg kg^−1^ of available phosphorus, and 378 mg kg^−1^ of available potassium was found in this soil. In the case of the pomegranate orchard, the soil presented a clay loam texture, pH of 8, an electrical conductivity of 0.6 dS m^−1^, and 1.1% of organic matter. A concentration of 9 mg kg^−1^ of available nitrogen, 13 mg kg^−1^ of available phosphorus, and 148 mg kg^−1^ of available potassium was found in this soil. Further details on the growing conditions of the walnut and pomegranate orchards can be found in Table 1.

### 2.2. Treatments

Because water stress is one of the main factors affecting the relationship between dendrometric variables and ETo [14], we applied four irrigation treatments to both species using a completely randomized design with three replicates using one tree as an experimental unit. For both species, the control treatment rewatered 100% of the ETo estimated by Penman–Monteith [35]. In the case of the walnut orchard, the treatments were 130% (T1), 60% (T2), and 50% (T3) of ETo, while treatments in the pomegranate orchard were 80% (T1), 60% (T2), and 40% (T3) of ETo. Differences in the watering regime between the species are explained by the specific irrigation problems observed at orchards level in these crops in Chile. For example, over-irrigation is still a problem in walnuts orchards, and we wanted to determine how this affects dendrometry data. Water was applied on a daily basis according to the evapotranspiration.

### 2.3. Meteorological Variables

To estimate hourly (EToh) and daily ETo using Penman–Monteith [35] as follows:

(1) $$ETo=\frac{∆\left(R_{n}-G\right)+\rho_{a}c_{p}\left(\delta e\right)g_{a}}{\left(∆+\gamma \left(1+\frac{g_{a}}{g_{s}}\right)\right)L_{v}}$$

where Δ is the rate of change of saturation specific humidity with air temperature (Pa K^−1^), $R_{n}$ is the net irradiance (Wm^−2^), G is ground heat flux (Wm^−2^), $\rho_{a}$ is air density (kgm^−3^), $c_{p}$ is specific heat capacity of air (Jkg^−1^K^−1^), δe is vapor pressure deficit (Pa), $g_{a}$ is conductivity of air (ms^−1^), γ is the psychrometric constant (Pa K^−1^), $g_{s}$ is stomata conductance or surface conductance (ms^−1^), and $L_{v}$ is the volumetric latent heat of vaporization (MJm^−3^). Hourly data of temperature, relative humidity, net radiation, wind speed, and rainfall were obtained from a meteorological station (Vantage Pro 2, Davis Instruments, Hayward, CA, USA). The pomegranate and walnut orchards were 900 m away from each other and meteorological data were obtained from the San Lorenzo station located at 6,409,089 S, 311,699 E, according to Datum WGS 84 H19, in the walnut orchard.

### 2.4. Dendrometry

Sensors of linear displacement (DD-S, Ecomatik, Dachau/Munich, Germany) were installed in the stems during the growing season without evident damage and with a representative vigor of the plant. Sensors were installed on the southern side of trees to avoid direct radiation and to minimize variability caused by specific locations. One sensor per tree replicate was installed; a total of 21 sensor were used in the study. Stem diameter fluctuations were recorded using a Dendrometer Data Logger (DL18, Ecomatik, Dachau/Munich, Germany), and from this data it was possible to calculate MDS, HDS, and EDS. In contrast to De la Rosa et al. (2016), we defined the beginning of the shrinkage as the time when the difference between the shrinkage value of one hour minus the earlier was negative. The HDS calculation included the first four hourly values after shrinkage start. 

### 2.5. Statistical Analysis

The analysis was separate for each species, using a completely randomized design with three replications. We used linear regression (LR) to model the relationship between EToh and the corresponding HDS. In addition, we used LR to analyze the differences in the relationships between hourly ETo and MDS/EDS. We verified the assumptions of all linear regression models (i.e., normal distribution, independence, and homoscedasticity) by visually checking the q–q and residual vs. fitted plots. To further improve the regression analysis and because stem shrinkage was observed to be highly dependent on ETo intensity, we categorized the daily relationship of HDS with time into four groups of slopes ranges that corresponded to categories of atmospheric demand during that day, and then we checked again the linear regression assumptions. 

## 3. Results and Discussion

### 3.1. Initial Linear Relationship between HDS and EToh 

**Figure 2:**
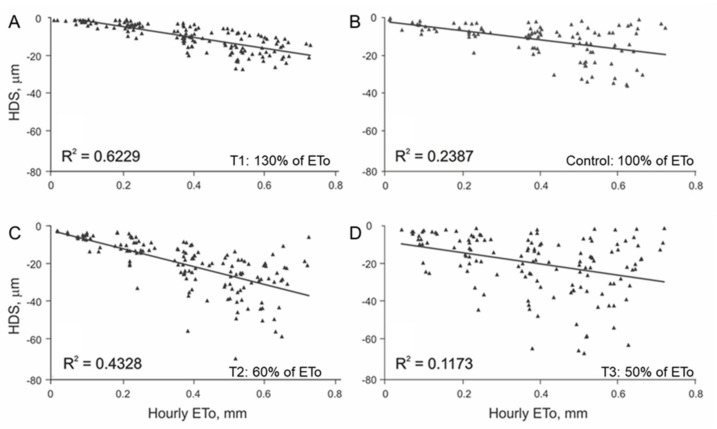
Relationship between EToh and HDS in walnut through the first four hours of branch shrinkage: (**A**) irrigation treatment T1; (**B**) irrigation treatment control; (**C**) irrigation treatment T2; (**D**) irrigation treatment T3. The solid line represents linear regression.

**Figure 3:**
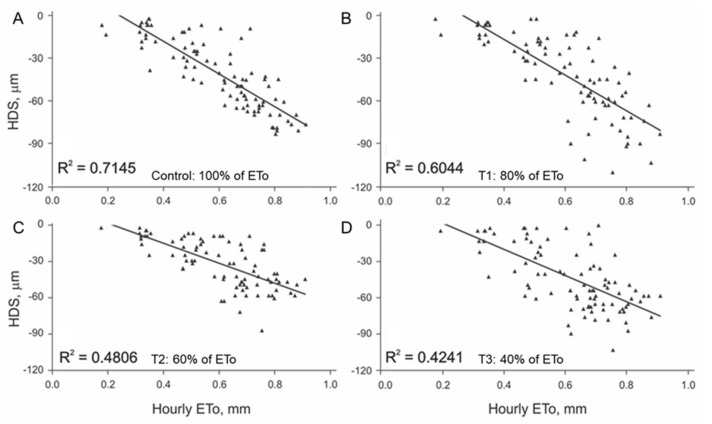
Relationship between EToh and HDS in pomegranate through the first four hours of branch shrinkage: (**A**) irrigation treatment control; (**B**) irrigation treatment T1; (**C**) irrigation treatment T2; (**D**) irrigation treatment T3. The solid line represents linear regression.

Through the first four hours of branch shrinkage, the relationship of HDS to EToh was similar for both species. Determination coefficients (*R*^2^) were higher than 0.6 for the irrigation treatments without deficit for walnut (Figure 2A,B) and pomegranate (Figure 3A). When water stress increased, the EToh to HDS relationship became weaker for walnut (Figure 2C,D) and for pomegranate (Figure 3C,D). These erratic readings of HDS that occurred with high values of EToh in water stressed plants might be an indication of hydraulic failure either in the xylem tissue and/or storage compartments such as dead fibers, nonfunctional vessels, and apoplastic pores [3]. To the best of our knowledge, it is not well understood how tissue hydration happens mechanistically within plants and across different species and studying this under field conditions is very challenging. Future experiments, including sap flow measurements along with real-time measured transpiration would be necessary to better understand dendrometry data.

### 3.2. Linear Regression Assumptions Analysis and Model Improvement

**Figure 4:**
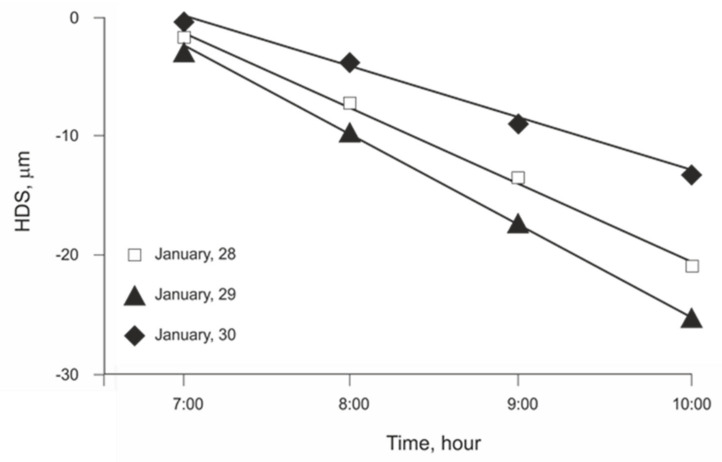
HDS through the first four hours of branch shrinkage over three days in walnut showing different slopes depending on the atmospheric conditions of the specific day.

A deeper analysis of the regression models (Figure 2 and Figure 3) shows that they do not meet the required assumptions for validation (normal distribution, independence, and homoscedasticity). Low values of EToh relate to high values of HDS, while HDS at higher values of EToh were erratic, meaning that the variance of the linear regression residuals increased with hourly evapotranspiration. This effect was evident in walnut and less for pomegranate. When searching for options to improve the models, we observed that the variation in the HDS values over time showed different intensities among days, leading to different slopes of HDS regarding time (Figure 4). The latter leads us to think that daily meteorological differences mainly explain this effect [22]. Based on this idea, we categorized the relationship of HDS with time into four groups of slopes ranges, separately for each species. To define the slope ranges, we kept the same number of cases in each slope group range. Afterward, we recalculated the linear regression model HDS to EToh (Table 2) for each HDS/time slope category. The resulting models showed that they meet all linear regression assumptions and that the values of *R*^2^ increased substantially for both species. Nevertheless, the magnitudes of daily slopes of HDS/time are different between species, which means that it is impossible to define a unique categorization for them (Table 2).

### 3.3. Daily ETo and MDS/EDS Relationship

The relationship between ETo and MDS and ETo and EDS was null for both species (Supplementary Materials Tables S1 and S2). For pomegranates, the results were very different from those described by Intrigliolo et al. [36]. They modeled daily MDS/ETo for pomegranate with *R*^2^ = 0.44. Fulton et al. [37] obtained a good result modeling MDS/*Ψ*_H_ with *R*^2^ = 0.74. Perhaps the crop phenological phase at the data collection time can explain the differences in results found in this study, since stem shrinkage can be less dependent on plant water status during summertime [13,20].

### 3.4. Significance of Our Findings

Here, we modeled HDS as a function of hourly evapotranspiration. The slope of the relationship between HDS versus time corresponded to the velocity at which the HDS occurred, which is directly related to the ETo intensity at that moment [25] and might be related to the plant capacity to keep transpiration at nearly the same velocity as ETo. Therefore, the categories of slope ranges corresponded to the categories of atmospheric demand during the considered period. Thus, we can define days with a high atmospheric demand and a highly negative slope, days with a moderate atmospheric demand and intermedia negative slopes, and days with a low atmospheric demand and slightly negative slopes [25]. To the best of our knowledge, this is the first study to report an extra step in the analysis to properly analyze this kind of data. Our findings call for a revision of these models and sets a new baseline for future analyses. Finally, the need for slope categorization shows that some additional variables are missing in this type of model. 

## Figures and Tables

**Table 1:** Details on the growing conditions of the walnut and pomegranate orchards.

	Walnut	Pomegranate
Orchard age	7 years	1 year
Planting density	7 × 7 m	5 × 5 m
Average plant height	5 m	3.5 m
Stem type measured	Growing season stem	Growing season stem
Tree phenological stage	Fruit ripening	Young vegetative grow
Measuring period	Summer, 38 days	Summer 29 days
Irrigation method	Micro sprinkler	Drip
Understory vegetation	Clean soil with mechanical weed removal	

**Table 2:** Linear regression models EToh/HDS through the first four hours of branch shrinkage, categorized by daily slope of HDS/time from the non-restricted irrigation treatment.

	Category	Slope_(HDS)_	b	m	*R* ^2^
Walnut	WS_1_	M_HDS_ > −4	2.5875	−19.643	0.86
Walnut	WS_2_	−4 > M_HDS_ > −5.5	2.6338	−30.208	0.87
Walnut	WS_3_	−5.5 > M_HDS_ > −6.5	3.9046	−36.796	0.91
Walnut	WS_4_	−6.5 > M_HDS_	3.4756	−43.851	0.86
Pomegranate	PS_1_	M_HDS_ > −14	11.0420	−85.0200	0.65
Pomegranate	PS_2_	−14 > M_HDS_ > −19	24.3540	−109.8300	0.78
Pomegranate	PS_3_	−19 > M_HDS_ > −22.5	40.8360	−130.0000	0.69
Pomegranate	PS_4_	−22.5 > M_HDS_	5.8220	−156.8500	0.83

Slope_HDS_ = daily slope HDS/time; b = intercept of the linear regression model; m = slope of the linear regression model.

## Data Availability

The data presented in this study are available upon request from the corresponding author.

## Supplementary Materials

The following are available online at https://www.mdpi.com/article/10.3390/plants11010031/s1: Table S1. Linear regression models ETo/MDS including all irrigation treatments: Table S2. Linear regression models ETo/EDS including all irrigation treatments.
