# Supplementary material for: Hourly Relationship between Reference Evapotranspiration and Shoot Shrinkage in Walnut Trees and Pomegranate under Deficit Irrigation
Source: Plants (Basel). 2021 Dec 23;11(1):31. doi: 10.3390/plants11010031 (PMC8747659; doi:10.3390/plants11010031)
Supplement: Supplementary file 1 [file plants-11-00031-s001.zip › plants-1491649-supplementary.pdf]

Supporting Information

Article title: Hourly relationship between reference evapotranspiration and shoot shrinkage in walnut trees and pomegranate
Authors: Eduardo Salgado, Nieggiorba Livellara, Esteban Chaigneau, Fernando Varas, Italo F. Cuneo

The following Supporting Information is available for this article:

Table S1 Linear regression models ETo / MDS including all irrigation treatments

	Irrigation treatment (%ETo)	Intercept b	Slope m	R^2
Walnut	130	140.71	-8.375	0.0166
Walnut	100	154.19	-7.073	0.0020
Walnut	60	533.88	-61.100	0.0566
Walnut	50	183.09	-3.073	0.0001
Pomegranate	100	182.80	15.156	0.0564
Pomegranate	80	109.96	23.898	0.0779
Pomegranate	60	203.80	1.507	0.0006
Pomegranate	40	119.38	24.480	0.1204

Table S2 Linear regression models ETo / EDS including all irrigation treatments

	Irrigation treatment (%ETo)	Intercept b	Slope m	R^2
Walnut	130	30.26	0.606	0.0004
Walnut	100	115.15	-13.153	0.0744
Walnut	60	192.11	-22.040	0.0601
Walnut	50	25.08	5.748	0.0029
Pomegranate	100	43.82	16.356	0.0134
Pomegranate	80	-118.29	41.569	0.0845
Pomegranate	60	-52.64	15.376	0.0401
Pomegranate	40	4.77	20.804	0.0169
